# Cardiac tamponade and para-aortic hematoma post elective surgical myocardial revascularization on a beating heart – a possible complication of the Lima-stitch and sequential venous anastomosis

**DOI:** 10.1186/1471-2261-14-72

**Published:** 2014-06-04

**Authors:** Anna Marcinkiewicz, Ryszard Jaszewski, Katarzyna Piestrzeniewicz, Radosław Zwoliński

**Affiliations:** 1 Cardiac Surgery Clinic, Chair of Cardiology and Cardiac Surgery, Military Medical Academy University Teaching Hospital - Central Veterans’ Hospital in Lodz, Sterling 1/3 St, Lodz 91-425, Poland; 2 Department of Cardiology, Chair of Cardiology and Cardiac Surgery, Military Medical Academy University Teaching Hospital - Central Veterans’ Hospital in Lodz, Lodz, Poland

**Keywords:** Lima-stitch, Cardiac tamponade, Aortic dissection

## Abstract

**Background:**

Off-pump coronary artery bypass (OPCAB) surgery can be associated with some intrinsic, but relatively rare complications. A pericardial effusion is a common finding after cardiac surgeries, but the prevalence of a cardiac tamponade does not exceed 2% and is less frequent after myocardial revascularization.

Authors believe that in our patient an injury of a nutritional pericardial or descending aorta vessel caused by the Lima stitch resulted in oozing bleeding, which gradually leaded to cardiac tamponade. The bleeding increased after introduction of double antiplatelet therapy and caused life-threatening hemodynamic destabilization. According to our knowledge it is the first report of such a complication after OPCAB.

**Case presentation:**

We present a case of a 61-year old man, who underwent elective surgical myocardial revascularization on a beating heart. On the 11th postoperative day the patient was readmitted emergently to the intensive care unit for severe chest pain, dyspnoea and hypotension. Coronary angiographic control showed a patency of the bypass grafts and significant narrowing of circumflex artery, treated with angioplasty and stenting. The symptoms and hemodynamic instability exacerbated. A suspicion of dissection of the ascending aorta and para-aortic hematoma was stated on 16-slice cardiac computed tomography. The patient was referred to the Cardiovascular Surgery Clinic. Transthoracic echocardiography revealed cardiac tamponade. On transesophageal echocardiography there were no signs of the ascending aorta dissection, but a possible lesion of the descending aorta with para-aortic hematoma was visualized. Emergent rethoracotomy and cardiac tamponade decompression were performed. 12 days after intervention the control 64-slice computed tomography showed no lesions of the ascending or descending aorta. On one-year follow-up patient is in a good condition, the left ventricular function is preserved and there is no pathology in thoracic aorta on echocardiography.

**Conclusions:**

Mechanical complications of surgical myocardial revascularization on a beating heart should be considered as a cause of the clinical and hemodynamic instability relatively early in the postoperative period. Echocardiographic examination must be the first step in diagnostics process in a patient after cardiac surgery.

## Background

Avoiding extracorporeal circulation (ECC) results in a lower inflammatory response, less myocardial or kidney damage and blood–brain barrier injury. Elimination of the ECC decreased the sex-depending differences in the results of surgical revascularization [1]. Still, there is no consensus about the long-term results of the beating heart surgery. Off-pump coronary artery bypass (OPCAB) surgery is associated with some intrinsic, but relatively rare complications: mechanical damage of the cardiac walls by suction stabilizers, coronary arteries injury by vascular loops or shunts, acute aortic dissection, lesions of the pulmonary vein, descending aorta or esophagus, gaseous embolism, as well as some exceptional adverse events as vasoplegic syndrome [2,3]. The crucial issue during revascularization on a beating heart allowing to perform anastomoses on the posterior and lateral wall is insertion of the pericardial stitch (firstly performed by Ricardo Lima) [4].

We presented a case of a patient who developed cardiac tamponade on the 11th postoperative day. Authors believe that it was a result of an injury of a nutritional pericardial or descending aorta vessel caused by the Lima stitch. A double antiplatelet therapy aggravated the bleeding. Echocardiography performed on the readmission could have allowed to make a proper diagnosis. According to our knowledge it is the first report of such a complication after OPCAB.

## Case presentation

**Figure 1:**
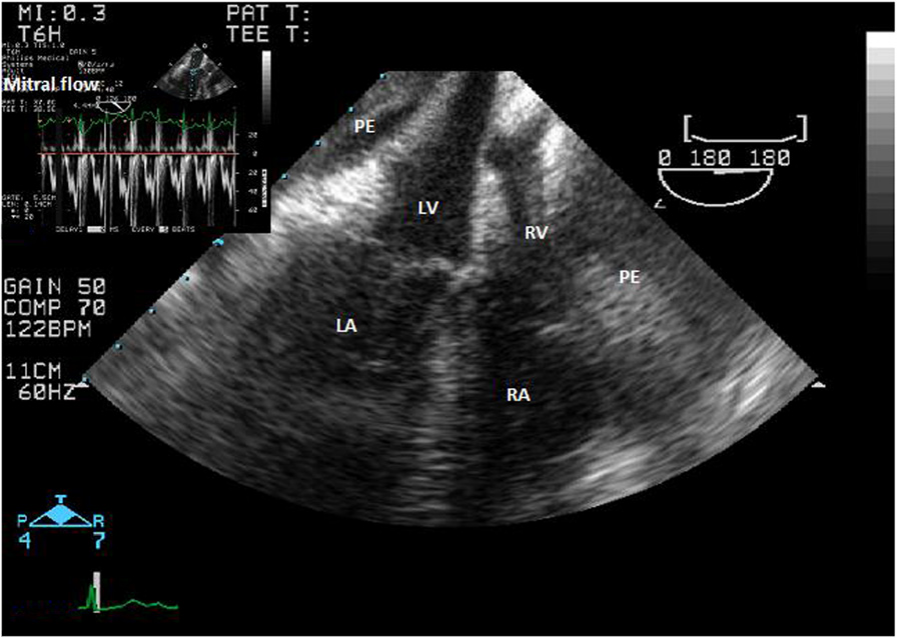
**Transesophageal echocardiography.** Cardiac tamponade. Pericardial effusion with collapse of left and right ventricles and significant respiratory variations of the transmitral flow velocities (left upper corner) – typical for cardiac tamponade. PE – pericardial effusion, LV - left ventricle, RV – right ventricle, LA - left atrium, RA – right atrium.

**Figure 2:**
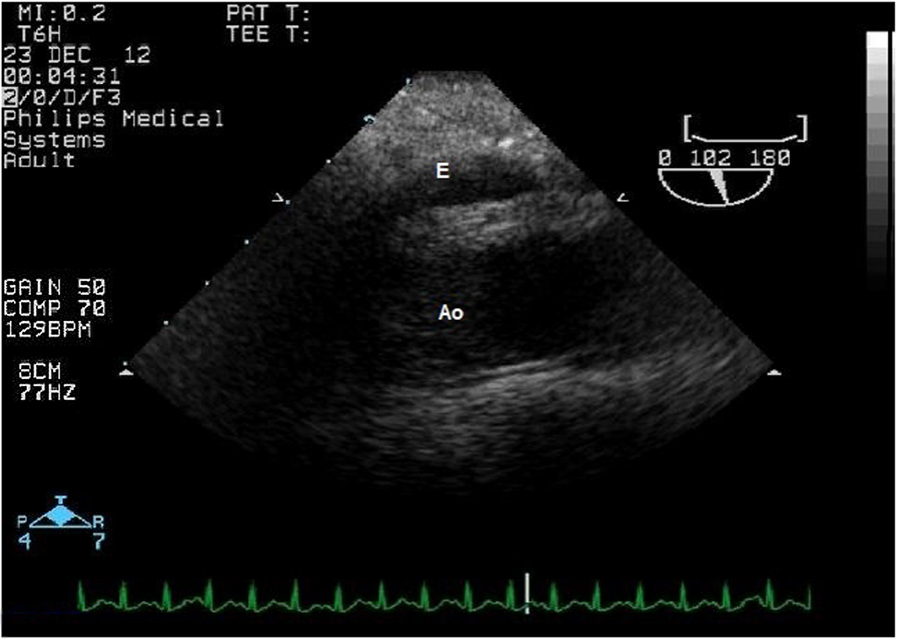
**Transesophageal echocardiography.** Descending aorta – long axis view. Increased distance between the probe and the aortic wall with the echo-free space suggestive for the para-aortic effusion. Ao – aorta, E - effusion.

A 61-year old man with a history of arterial hypertension with a 3-month history of unstable angina was admitted to hospital. Coronary angiography revealed significant bi-level stenosis of the left anterior descending artery (LAD) and 70% stenosis in the central segment of circumflex artery (Cx) with its further amputation. The patient was referred to elective myocardial surgical revascularization on a beating heart, without extracorporeal circulation. Left internal mammary artery (LIMA) was used to bypass the left anterior descending artery (LAD) and the sequential venous anastomosis to diagonal and marginal branches was performed. A pericardial deep stitch (Lima stich) allowed to perform anastomoses on the lateral cardiac wall. The surgery and the postoperative period was uneventful. Patient was extubated the same day and discharged in a good condition 7 days later. Early postoperative transthoracic echocardiography (TTE) revealed mild hypokinesis of the para-apical and central segments of the interventricular septum and basal segments of the infero-posterior wall with preserved ejection fraction (EF) - 54% and small pericardial effusion (0,9 cm).On the 11th postoperative day the patient was readmitted to the intensive care unit in a local hospital for severe chest pain, dyspnoea NYHA IV functional class and hypotension. The blood pressure was 80/50 mmHg and the heart rate was 120/min. On electrocardiogram (ECG) nonspecific changes of ST-segment in left precordial leads were observed (1 mm downsloping depression, with flat/slight negative T waves in I, aVL, V4-V6). The diagnosis of an acute coronary syndrome without persistent ST-segment elevation was made. Mild elevation in cardiac enzymes was detected. The arterial pressure was maintained with catecholamines infusion. Coronary angiographic control showed patent LIMA and sequential venous grafts. Significant narrowing of circumflex artery which was treated with angioplasty and stenting with drug-eluting stent (DES) was also found. Consequently a double antiplatelet therapy was commenced. The symptoms and hemodynamic instability even exacerbated. 16-slice computed tomography (CT) disclosed effusion in the pericardial sac and around the ascending aorta what was suggestive for the dissection of the anterior wall of the ascending aorta. Patient in severe clinical condition was emergently referred to the Cardiovascular Surgery Clinic in Lodz where mechanical ventilation was applied for the cardiorespiratory insufficiency not controlled with catecholamines infusion. The blood tests showed increased level of troponin T (119,3 ng/L), leukocytes count (21.700 mc/L), platelets count (453.000 mc/L), C-reactive protein (201,3 mg/L), significantly increased liver enzymes and high creatinine level suggesting multiple organ dysfunction syndrome (MODS). Creatine kinase-MB was within normal limits (18 U/L). The clinical presentation together with TTE/TEE allowed to diagnose cardiac tamponade. There were no echocardiographic signs of dissection of the ascending aorta, but an increased distance between the probe and the aortic wall was suggestive for the para-aortic effusion (mediastinal hematoma) (Figure 1). A slight flow from the descending aorta towards the effusion was noticed suggesting either the ostial flow in the pericardial or esophageal aortic branch or the aortic rupture (Figure 2).

Patient was emergently transferred to the operating theatre, where rethoracotomy was performed. Blood from the pericardial sac was evacuated and cardiac tamponade decompressed with dramatic improvement in clinical status of the patient. Para-aortic hematoma was confirmed but there was no evidence of damage to the aorta. The drainage from pericardial sac was 270 ml during the following day.

12 days after intervention the control 64-slice CT showed no lesions of the ascending or descending aorta. On one-year follow-up patient is in a good condition, the left ventricular function is preserved and there is no pathology in thoracic aorta on TEE.

## Discussion

The presented case requires differential diagnosis of several possible causes of the significant hemodynamic decompensation that occurred on the 11th postoperative day.

Early graft occlusion and myocardial infarction was one of the considered causes of patient’s symptoms and hemodynamic instability. Prevalence of this complication is estimated on 3-12% for venous grafts and 1–2,5% for the internal thoracic artery [5]. Vascular loops and tourniquets passed under the coronary arteries may damage arterial walls and intraluminal shunts may cause lesions of the vascular endothelium. Although pericardial effusion is a common finding after cardiac surgeries, but the prevalence of a cardiac tamponade does not exceed 2% and is less frequent after myocardial revascularization [6]. The effusion or hematoma is usually not massive and is more likely regional than circumferential. That is why the role of fluid accumulated in pericardium that should be considered as a cause of severe clinical condition and cardiac tamponade is neglected. The final clinical outcome depends on the appropriate time of rethoracotomy [7].

Dressler’s syndrome was considered but during rethoracotomy not a bloody effusion but hematoma and fresh blood filling the pericardial sac were found. It seemed apparent that bleeding was a cause of the cardiac tamponade.

Unfortunately TTE, that should be the first step of diagnostic process in this particular case and might have disclosed pericardial effusion was not performed on admission in the local hospital. At that time, mild elevation of troponin T level was not diagnostic for an acute coronary event, as blood levels of this marker may remain increased up to 2 weeks after surgical myocardial revascularization. Cardiac tamponade was another possible cause of the elevation of troponin level. In our opinion stenting of the circumflex artery was not appropriate as the marginal venous bypass graft was well functioning. Introduction of double antiplatelet therapy might have increased the oozing bleeding and caused further patient’s destabilization.

Another possible complication after beating heart surgery is an intraoperative mechanical heart wall damage with suction devices. Mandke et al. [8] presented a case of subepicardial hematoma, which was evacuated intraoperatively, but early after the surgery dissecting intramural hematoma with tamponade occurred.

OPCAB procedure may lead to an acute ascending aorta dissection as a result of the partial clamping of the aorta for suturing the proximal anastomoses. Hagl and Griepp [9] suggested that in case of poor aortic wall quality the risk of dissection is even higher for OPCAB than procedures under ECC. Some authors emphasized underestimation of this complication, which prevalence is 3-5%, especially in cases of sudden postoperative deaths with ventricular tachyarrhythmias [10]. Iatrogenic ascending aorta dissection can occur at any time after the operation. It often presents as acute neurological deficits, rapidly arising mediastinal hematoma and finally aortic rupture. Such cases must be aggressively treated. However iatrogenic dissection can be clinically silent and found incidentally with conservative treatment as a solution [10]. In our patient neither the clinical presentation nor the dynamics of arising effusion indicated dissection. The diagnosis of our experienced echocardiographist based on TEE excluded the presence of aortic dissection and was definitively confirmed during re-exploration of the mediastinum.

The most interesting issue in this case are the confusing results of imaging examinations. The firstly performed 16-slice CT, considered as diagnostic tool of relatively low sensitivity and specificity, revealed limited dissection of the ascending aorta at the area of the proximal bypass graft anastomosis, which was not confirmed on subsequent TEE. Thinking of the possible explanation of this diagnostic discrepancy we considered iatrogenic aortic dissection as a result of proximal bypass graft anastomosis or at the time of coronary artery bypass angiography, self-healed within following hours*.* Garg P et al. [11] described spontaneous recovery of aortic dissection in several hours.

Salerno et al. [12] reported a fatal case of descending aorta injury. Fukui et al. [13] described a case of a lesion in the pulmonary vein with retrocardiac hematoma formation.

The presented case is an example of a rare but extremely dangerous complication of beating heart revascularization. The authors believe that a lesion of a pericardial or aortic nutritional vessel caused by Lima-stitch resulted in the life-threatening complication. Echocardiographic examination remains the first step in diagnostic process in a patient after cardiac surgery.

## Conclusions

Mechanical complications of surgical myocardial revascularization on a beating heart should be considered as a cause of the clinical and hemodynamic instability relatively early in the postoperative period.

## Consent

Written informed consent was obtained from the patient for publication of this Case report and any accompanying images. A copy of the written consent is available for review by the Editor of this journal.

## Abbreviations

DES: Drug-eluting stent; CT: Computed tomography; TTE: Transthoracic echocardiography; TEE: Transesophageal echocardiography; ECC: Extracorporeal circulation; OPCAB: Off-pump coronary artery bypass; LAD: Left anterior descending artery; Cx: Circumflex artery; LIMA: Left internal mammary artery; EF: Ejection fraction; ECG: Electrocardiogram; MODS: Multiple organ dysfunction syndrome.

## Competing interest

The authors declare that they have no competing interests.

## Authors’ contributions

AM made the conception and design of the paper, drafted the manuscript, was responsible for acquisition of the data, literature searching, interpretation of the data and its revision. RJ helped to revise it critically for important intellectual content, participated in its design and coordinated. KP prepared the echocardiograms, helped to draft the manuscript, helped to revise it critically for important intellectual content. RZ helped to draft the manuscript, helped to design it properly and choose appropriate literature, helped to revise it critically for important intellectual content. All authors were involved in the patient’s treatment and made a substantial contribution to the manuscript preparation. All authors read and approved the final manuscript.

## Pre-publication history

The pre-publication history for this paper can be accessed here:

http://www.biomedcentral.com/1471-2261/14/72/prepub
